# Determination of clusters and factors associated with dengue dispersion during the first epidemic related to *Dengue virus* serotype 4 in Vitória, Brazil

**DOI:** 10.1371/journal.pone.0175432

**Published:** 2017-04-07

**Authors:** Creuza Rachel Vicente, Karl-Heinz Herbinger, Crispim Cerutti Junior, Camila Malta Romano, Aline de Souza Areias Cabidelle, Günter Fröschl

**Affiliations:** 1 Center for International Health, Medical Center of the University of Munich (LMU), Munich, Germany; 2 Division of Infectious Diseases and Tropical Medicine, Medical Center of the University of Munich (LMU), Munich, Germany; 3 Department of Social Medicine, Federal University of Espírito Santo, Vitória, Brazil; 4 Institute of Tropical Medicine, LIM-52 (LIMHC), University of São Paulo, São Paulo, Brazil; 5 Coordination of Epidemiological Surveillance, Department of Health, Vitória, Brazil; Centro de Pesquisas René Rachou, BRAZIL

## Abstract

Dengue occurrence is partially influenced by the immune status of the population. Consequently, the introduction of a new *Dengue virus* serotype can trigger explosive epidemics in susceptible populations. The determination of clusters in this scenario can help to identify hotspots and understand the disease dispersion regardless of the influence of the population herd immunity. The present study evaluated the pattern and factors associated with dengue dispersion during the first epidemic related to *Dengue virus* serotype 4 in Vitória, Espírito Santo state, Brazil. Data on 18,861 dengue cases reported in Vitória from September 2012 to June 2013 were included in the study. The analysis of spatial variation in temporal trend was performed to detect clusters that were compared by their respective relative risk, house index, population density, and income in an ecological study. Overall, 11 clusters were detected. The time trend increase of dengue incidence in the overall study population was 636%. The five clusters that showed a lower time trend increase than the overall population presented a higher incidence in the beginning of the epidemic and, compared to the six clusters with higher time trend increase, they presented higher relative risk for their inhabitants to acquire dengue infection (*P*-value = 0.02) and a lower income (*P*-value <0.01). House index and population density did not differ between the clusters. Early increase of dengue incidence and higher relative risk for acquiring dengue infection were favored in low-income areas. Preventive actions and improvement of infrastructure in low-income areas should be prioritized in order to diminish the magnitude of dengue dispersion after the introduction of a new serotype.

## Introduction

Dengue is endemic in approximately 120 countries located in tropical and subtropical areas [1]. Infections are estimated to occur in 390 million individuals globally per year, considering symptomatic and asymptomatic cases [2]. *Dengue virus* (DENV) presents four serotypes that are antigenically distinct. Therefore, an infection by a certain serotype does not confer lifelong immune protection against the other serotypes [3]. A recent study also presented cases with reinfection by the same serotype, opposing the previous idea of lifelong protection to homotypic reinfection [4]. The immunity profile of a given population affects partially the magnitude of dengue occurrence, thus allowing explosive epidemics when a new serotype is introduced in a naive and therefore highly susceptible population. The epidemics weaken in the course of time due to the depletion of the susceptible population [5, 6].

The presence of the *Aedes* mosquito, the vector of dengue, also influences the transmission intensity and is related to climatic conditions and to the availability of breeding sites constituted by stagnant water. Breeding site formation is favored by the lack of basic sanitation, garbage collection, water supply, and inadequate water storage [7–9]. Urban areas meet requirements for intense dengue transmission, providing conditions for breeding in proximity to human habitats with a high population density (PD) [7, 10, 11]. Adult mosquitoes stay near to their breeding sites, with longer flights depending on the availability of feeding and reproduction sources. The average flight distance is lower than 150 meters [12], resulting in usually very localized transmission.

In Brazil, the reemergence of dengue epidemics occurred in 1980’s [13], when an outbreak of *Dengue virus* serotype 1 (DENV-1) and *Dengue virus* serotype 4 (DENV-4) was registered in Roraima state [14]. In 2010, DENV-4 was detected in the North of Brazil after more than 20 years without it being reported in the country. In the following years, this serotype disseminated to other Brazilian regions [15], triggering an epidemic with approximately 2 million cases in 2013.

In the city of Vitória, the capital of Espírito Santo state, Brazil, the first infection by DENV was detected in 1995 [16]. In April 2012, the first case of DENV-4 was reported in the city. The incidence of dengue in Vitória increased after September 2012, resulting in an unprecedented epidemic in 2013, with 19,449 reported suspect cases.

The determination of clusters is a relevant tool in order to understand the dynamics of dispersion of any infectious disease, enabling the identification of hotspots and helping to improve the disease control. The present study evaluated the spread of dengue during the first circulation of DENV-4 in Vitória, and the factors that might influence the dispersion pattern.

## Materials and methods

### Study design

An analysis of spatial variation in temporal trend was performed to detect clusters. The study comprised 18,861 cases of dengue detected in public and private health services and reported to the Health Department of Vitória, from September 2012 to June 2013. The cases were confirmed by laboratory (n = 3,801; 20.2%) or clinical-epidemiological criteria (n = 15,060; 79.8%). Determination of the serotype was possible in 74 cases (0.4%), and all were caused by DENV-4. This period of ten months was selected in order to comprise the immediate epidemic after the first detection of DENV-4 in Vitória, in April 2012. An ecological study was applied to evaluate factors associated with dengue dispersion.

### Study area

Vitória is the capital of Espírito Santo state, located on the coast of the Brazilian Southeast. A census in 2010 defined Vitória with an area of approximately 98 km^2^ and a population of about 319,000 inhabitants [17]. In Vitória, the climate is tropically humid, with an annual average temperature of approximately 24°C and an annual average precipitation of 1,153 mm [18]. The rainy season occurs annually between October and March, corresponding to spring and summer seasons.

### Data source

Data on the home addresses of reported cases were accessed through the Information System for Notifiable Diseases (SINAN) (http://sinan.saude.gov.br/). The Center of Surveillance in Environmental Health of Vitória provided data on House Index (HI) [19, 20]. Data on population number, area in square kilometers (km^2^) and average income in the districts were obtained from the Census 2010, performed by the Brazilian Institute of Geography and Statistics [17].

### Definitions of terms and variables

Dengue case corresponds to a patient with acute febrile illness lasting up to seven days, accompanied by at least two of the following signs or symptoms: headache, retro-orbital pain, myalgia, arthralgia, prostration, malaise or rash associated or not with bleedings or hemorrhages, confirmed by laboratory or clinical-epidemiological criteria [21]. A cluster is defined as a spatial conglomerate of dengue cases, where the time trend increase (TTI) inside is different from the TTI outside [22]. TTI is defined as the relative increase of dengue incidence inside and outside the cluster on the log-linear scale, being the percent increase constant over time [22]. Location of the dengue case is defined as the address of residence as indicated by the patient in the report form. Districts are intra-urban subdivisions legally established by laws of the municipal assembly and sanctioned by the city mayor. Income is the monthly average remuneration per capita of inhabitants living in city districts, in Brazilian currency (Reais). PD is the total number of inhabitants living in a certain district, divided by the area of the district, in square kilometers (inhabitants/km^2^). HI is the number of buildings where larvae of *Aedes aegypti* could be identified, divided by the number of buildings evaluated, expressed as a percentage [23, 24]. HI is a parameter to measure the presence of a vector population, and by extension, the potential for dengue transmission. HI evaluates the presence of larvae in buildings randomly selected. The sampling involved districts included in strata based on social and environmental similarities between them. The HI value of a district was considered the same value of the HI in the strata to which it belonged in the survey performed by the Center of Surveillance in Environmental Health of Vitória. The HI of a cluster is defined as the median of the HI of the districts that compose the cluster. An HI lower than 1% is considered to correspond to a low potential for dengue transmission, between 1% and 3.9% to a medium potential and higher than 3.9% to a high potential [24]. The values of HI used in the study were those available for the period, corresponding to the months of October 2012 and March 2013.

### Geocoding

The plugin MMQGIS of the software QGIS 2.8.2-Wien was used to geocode the dengue cases using data on residence address. This plugin uses Google Maps ™ API to geocode the addresses, and the result is a point shapefile. The shape of a map presenting the limits of the districts from the site Geoweb Vitória (www.geoweb.vitoria.es.gov.br) was used to check the position of the cases geocoded.

### Analysis

The software SatScan version 4.9 was used for the analysis of spatial variation in temporal trend in a period of 10 months for detecting clusters. The central position of the district was applied as a reference for case locations, determining latitude and longitude. The maximum cluster size was defined as 1 km of diameter in a circular shape. The scan statistic applies the discrete Poisson probability model and uses a spatial scanning window to calculate the TTI inside, outside and for each location and size of the scanning window. This scan statistic identifies clusters with a TTI higher than the TTI outside the cluster and presents the result in percent increase on a log-linear scale over time. The null hypothesis is that all TTI are similar, being a base for the analysis of log likelihood ratio (LLR). LLR corresponds to the unlikeliness of differences between the TTI occurring due to chance [22]. A *P*-value lower than 0.05 and an LLR higher than 6.92 define a significant cluster. The LLR is defined by the following formula:

$$LLR={\left(\frac{N}{Ec/\left[n\right]}\right)}^{n}{\left(\frac{N-n}{N-Ec[n]}\right)}^{N-n}I()$$ (1)

N = Total number of cases

n = Number of cases inside the window

Ec[n] = Expected number of cases inside the window under the null hypothesis

I() = Indicator function with a value equal to 1 for all windows

The expected number of cases inside the window is equal to the total number of observations, times the size of scanning window, divided by the size of the total study area. Under the null hypothesis, the number of expected cases in each area is directly proportional to its population size [22].

The expected number of cases outside the window is equal to the total number of cases observed minus the expected number of cases inside the window under the null hypothesis [22].

SatScan also used the method defined by Kulldorff [22] to calculate the relative risk (RR) for acquiring dengue, dividing the estimated risk inside the cluster by the estimated risk outside the cluster. The RR is defined by the following formula:

$$RR=\frac{n/Ec(n)}{\left(N-n\right)/\left(Ec[N]-Ec[n]\right)}=\frac{n/Ec[n]}{\left(N-n\right)/\left(N-Ec[n]\right)}$$ (2)

N = Total number of cases in the dataset

n = Number of cases observed in the cluster

Ec[n] = Expected number of cases inside the cluster under the null hypothesis

Ec[N] = Total number of cases observed

The clusters were grouped into lower and higher TTI, considering the TTI of the overall study population as the cutoff. The use of this value (635.85) as a cutoff considers its proximity with the values of TTI outside all clusters, which ranged from 564.49 to 748.03. The closeness of these values occurs because the TTI outside the cluster considers almost the overall study population, only excluding the population inside the cluster. Therefore, the cutoff does not generate implications in the grouping of the clusters, even with the LLR analysis considering the TTI outside the cluster as a parameter to define a significant cluster. In the software R version 3.0.1 (www.r-project.org), the groups with lower and higher TTI were compared regarding PD, income, HI in October 2012, HI in March 2013, HI difference, and RR for dengue acquisition, using Mann-Whitney U-test. The level of significance has been set to 0.05.

Kernel method was applied to construct heat maps, using the exact location of the cases reported and configured with an area of 1 km of diameter in the software QGIS 2.8.2-Wien. The Kernel map was presented in order to illustrate the fluctuation of a total number of new cases reported in each individual area per month. It does not permit a comparison of the force of the infection between different clusters since the method does not consider differences in the base population. In order to provide the information of the force of the infection, the dengue incidence per month in each cluster was calculated in the software Microsoft Excel 2013 and displayed in a graph.

### Ethical aspects

The study protocol was approved by the Research Ethics Committee of the Health Sciences Center at the Federal University of Espírito Santo (opinion number 881,909) and the Ethics Committee of the University of Munich (opinion number 231–15). All data were analyzed anonymously.

## Results

**Fig 1:**
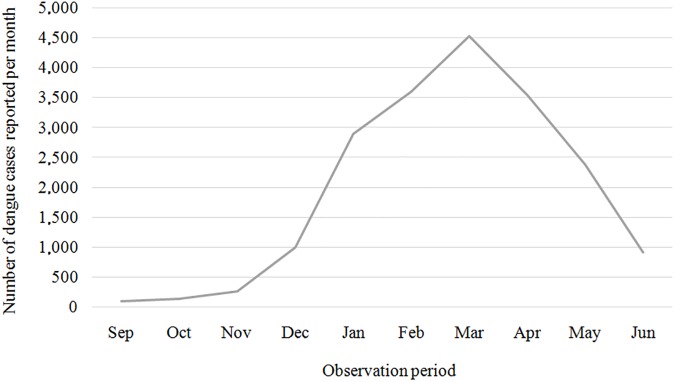
Cases of dengue reported in Vitória per month, September 2012 to June 2013.

From September 2012 to June 2013, the health services in Vitória reported 19,397 dengue cases to the Health Department of the municipality, of which 18,861 (97.3%) were geocoded based on the availability of data on the address of residence and the matches with the database of Google Maps ™ API. Among these cases, 18,770 (99.5%) were geocoded in street level and 91 (0.5%) in district level. The range of monthly incident cases of dengue for the entire catchment area varied from 105 in September 2012 to 4,529 in March 2013 (Fig 1).

**Fig 2:**
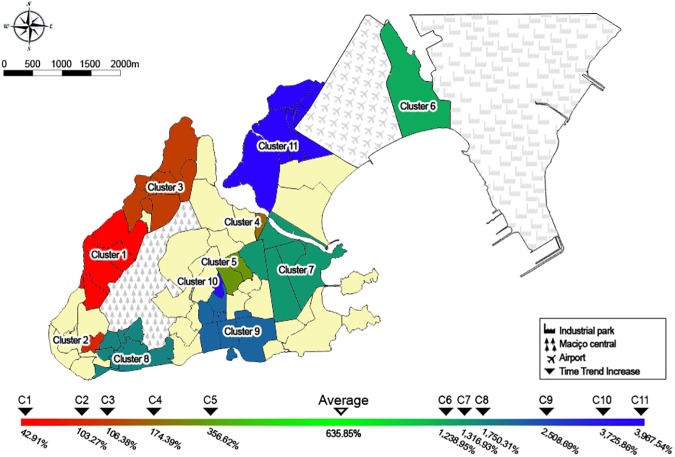
Clusters with lower or higher time trend increase in Vitória, September 2012 to June 2013. TTI—Time trend increase: incidence increase over the time.

Scan analysis detected 11 clusters: cluster 1 (LLR = 112.00, *P*-value <0.01), cluster 2 (LLR = 12.00, *P*-value <0.01), cluster 3 (LLR = 94.81, *P*-value <0.01), cluster 4 (LLR = 9.69, *P*-value <0.01), cluster 5 (LLR = 8.73, *P*-value = 0.01), cluster 6 (LLR = 11.65, *P*-value <0.01), cluster 7 (LLR = 12.77, *P*-value <0.01), cluster 8 (LLR = 20.66, *P*-value <0.01), cluster 9 (LLR = 36.17, *P*-value <0.01), cluster 10 (LLR = 9.95, *P*-value <0.01), and cluster 11 (LLR = 78.70, *P*-value <0.01). The TTI in Vitória as a whole was 635.85%. Five clusters presented a lower TTI than the overall study population (clusters 1 to 5), varying from 42.91% (cluster 1) to 356.62% (cluster 5). In clusters with lower TTI, the RR ranged from 1.26 (cluster 3) to 3.05 (cluster 4). The HI in October 2012 varied from 0.7 (cluster 4) to 3.0 (clusters 2 and 5), and in March 2013 the HI varied from 0.9 (cluster 3) to 3.0 (cluster 5). Clusters 2 and 3 presented a reduction in the HI from October 2012 to March 2013, the HI remained similar in cluster 5, and the HI increased over time in clusters 1 and 4 (Fig 2 and Table 1).

**Table 1:** Spatial variation in temporal trends for clusters with lower (cluster 1 to 5) and higher (cluster 6 to 11) time trend increase in Vitória, from September 2012 to June 2013.

	Cluster 1	Cluster 2	Cluster 3	Cluster 4	Cluster 5	Cluster 6	Cluster 7	Cluster 8	Cluster 9	Cluster 10	Cluster 11	Vitória
Population	16,226	2,664	26,232	2,031	12,709	39,157	35,190	15,568	14,001	1,850	26,423	319,031
TTI inside (%)	42.91	103.27	106.38	174.39	356.62	1,238.95	1,316.93	1,750.31	2,508.69	3,725.86	3,967.54	635.85
TTI outside (%)	748.03	650.22	759.40	651.04	664.58	605.83	605.73	601.08	591.33	626.09	564.49	N.A.
PD (inhabitants/km^2^)	6,516	14,021	8,862	14,507	20,498	15,003	9,802	9,610	6,223	14,231	6,689	3,338*
Income (reais)	662	538	514	620	652	2,260	2,607	1,136	1,506	683	1,394	
HI October 2012 (%)	1.4	3.0	1.6	0.7	3.0	0.7	1.8	2.8	2.2	2.2	1.2	N.A.
HI March 2013 (%)	2.7	2.1	0.9	1.6	3.0	1.5	6.2	6.4	5.6	3.7	2.6	N.A.
HI difference (%)	48	-43	-78	56	0	53	71	56	61	41	54	N.A.
Cases observed	1,384	261	1,915	362	1,361	1,279	1,184	1,005	973	172	1,209	18,861
Incidence	8,530	9,797	7,300	17,824	10,709	3,266	3,365	6,456	6,950	9,297	4,576	N.A.
RR	1.48	1.67	1.26	3.05	1.87	0.52	0.54	1.10	1.19	1.58	0.76	N.A.

PD—Population density, HI—House index: the relation between the number of buildings with larvae of *A*. *aegypti* and the number of buildings evaluated, RR—Relative risk: estimated risk inside the cluster divided by the estimated risk outside the cluster, TTI—Time trend increase: incidence increase over the time. Incidence: number of cases observed in the cluster from September 2012 to June 2013, divided by the population of the cluster, multiplied by 100,000. N.A.—Not Applicable.

*Data corresponds to the situation before the State Law 9,972 applied in 2012, which redefined the boundaries of Vitória, excluding three districts that were considered in the Census 2010.

Six clusters presented a higher TTI than the overall study population (clusters 6 to 11), varying from 1,238.95% (cluster 6) to 3,967.54% (cluster 11). The RR in these six clusters ranged from 0.52 (cluster 6) to 1.58 (cluster 10). The HI in October 2012 varied from 0.7 (cluster 6) to 2.8 (cluster 8), and in March 2013 the HI varied from 1.5 (cluster 6) to 6.4 (cluster 8). In all these clusters, the HI increased over time (Fig 2 and Table 1).

Overall, the RR for dengue acquisition was higher in the clusters with lower TTI than in the clusters with higher TTI. Clusters with lower TTI also presented lower incomes than clusters with higher TTI. PD and HI were not significantly different between clusters with lower and higher TTI (Table 2).

**Table 2:** Comparison of clusters with lower and higher time trend increase.

	Lower TTI (clusters 1 to 5) Median (interquartile range)	Higher TTI (clusters 6 to 11) Median (interquartile range)	*P*-value*
PD (inhabitans/km^2^)	14,021 (8,862–14,507)	9,706 (6,689–14,231)	0.66
Income (reais)	620 (538–652)	1,450 (1,136–2,260)	<0.01
HI October 2012 (%)	2.0 (1.0–3.0)	2.0 (1.0–2.0)	0.79
HI March 2013 (%)	2.1 (1.6–2.7)	4.65 (2.6–6.2)	0.13
HI difference (%)	0 (-43–48)	55 (53–61)	0.05
RR	1.67 (1.48–1.87)	0.93 (0.54–1.19)	0.02

TTI—Time trend increase: incidence increase over the time, PD—Population density, HI—House index: the relation between the number of buildings with larvae of *A*. *aegypti* and the number of buildings evaluated, RR—Relative risk: estimated risk inside the cluster divided by the estimated risk outside the cluster.

* Mann-Whitney U-test.

**Fig 3:**
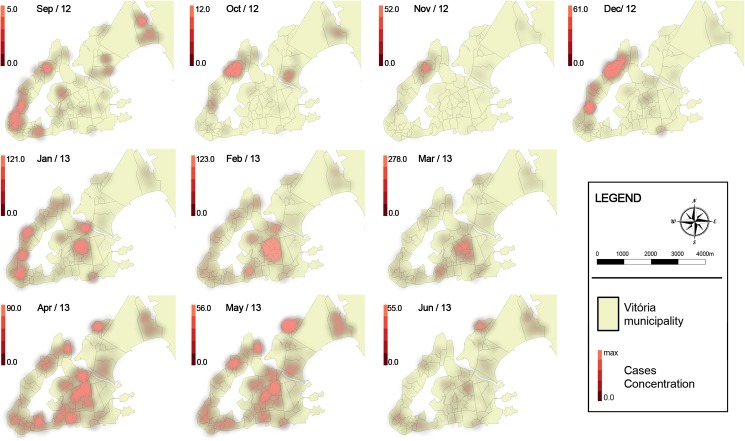
Dengue dispersion during an epidemic in Vitória, September 2012 to March 2013. N.B.: The color gradients vary from the minimum to the maximum number of dengue cases reported in the respective month per an area of 1 km of diameter.

**Fig 4:**
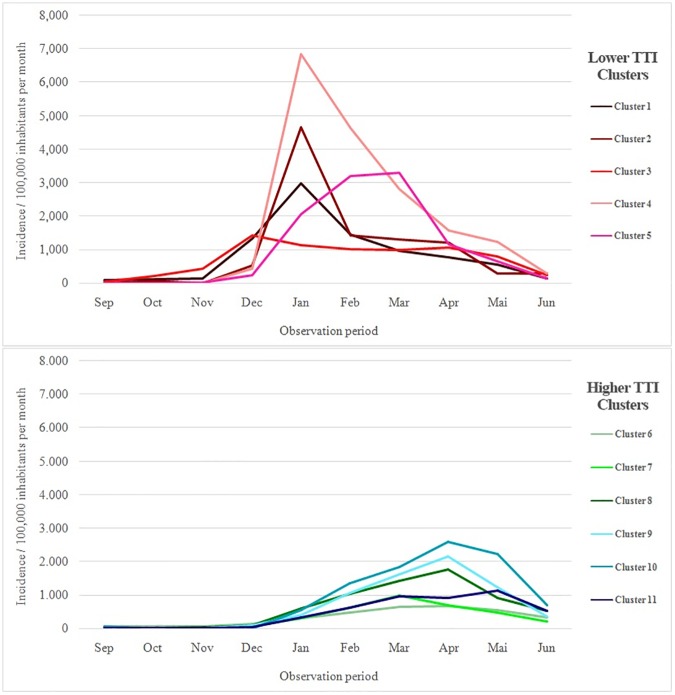
Dengue incidence in the clusters in Vitória, September 2012 to June 2013.

At the beginning of the epidemic, dengue occurrence was more concentrated in the clusters 1 and 3, followed by the clusters 2, 4 and 5 in January 2013. After February 2013 the disease affected larger areas, increasing its occurrence in clusters with higher TTI, and notably at the end of the period studied (after April 2013) in the cluster 11 (Fig 3). The incidence per month in the clusters also evidenced the earlier and higher occurrence of dengue in clusters with lower TTI, and a late increment of dengue occurrence in clusters with higher TTI (Fig 4).

## Discussion

The present study assessed the dispersion of dengue after the first introduction of DENV-4 in the highly susceptible population of Vitória, Brazil. Furthermore, the study evaluated the influence of factors, such as HI, PD, and income, in the dispersion pattern of the disease. The epidemic was more intense during the warm and rainy season, from January 2013 to March 2013, similar to the dengue seasonal pattern in previous years. During this epidemic, clusters with lower TTI presented a higher RR for dengue acquisition than clusters with higher TTI. In clusters with lower TTI, the incidence was explosive in the beginning of the epidemic and did not suffer a high increase over the time compared to clusters with higher TTI.

In these clusters with lower TTI, the limited increase in incidence over the time possibly occurred due to the depletion of susceptible individuals, since four of these clusters (clusters 1, 2, 4, and 5) remained with a medium potential for dengue transmission according to the HI in March 2013. It corroborates the observations of Medronho [25] and Chadee [6], in whose opinion the modulation of the epidemic process has been occurring in Brazil due to the immunity of the population and not due to vector control programs. Residents of clusters with lower TTI presented lower income than those living in clusters with higher TTI. Generally, low-income areas present poor infrastructure with a deficient garbage collection and poor provision of tap water, which in turn lead to water storage for human consumption in barrels and other recipients, contributing to the presence of breeding sites. These types of water storages were found in 12.5% of breeding sites in cluster 2, 28.6% - 60% in cluster 3 and 35.7% in cluster 5, in October 2012.

Despite the income and RR contrast, the HI difference between clusters with lower and higher TTI was not significant in any period. It was not possible to conclude whether the sampling for HI calculation affected the results, for example by not describing adequately the entomological scenario in areas with lower TTI. It has to be noted that HI has previously been reported to be an inaccurate measurement of mosquito procreation and an imprecise indicator of potential of dengue transmission [5]. Some studies have found relations between higher larval density and increased dengue occurrence [7, 26–28] whereas other studies showed no relation [6, 29–31].

Clusters with higher TTI presented an HI increment from October 2012 to March 2013, although not significantly different from the clusters with lower TTI. Nevertheless, according to HI in March 2013, three clusters with higher TTI presented a high potential for dengue transmission (clusters 7, 8 and 9), and three presented medium potential (clusters 6, 10 and 11). Probably, a higher density of vectors in this period contributed to an increasing incidence. Interestingly, populations living in clusters with higher TTI presented, overall, low RR for acquiring dengue infection despite the elevated HI. The PD in these clusters was not significantly different from clusters with lower TTI. Possibly, the poor housing conditions in low-income areas increase the contact of the population with the vector, and consequently the dengue transmission.

Socioeconomic conditions seem to interfere with the occurrence of dengue infection even within clusters with higher TTI. Thereby, the cluster 10 presented the highest RR among those with higher TTI, being the one among them with a lower income level. Previous studies evaluated socioeconomic variables, relating them to dengue occurrence. Some authors demonstrated an association of dengue occurrence with low socioeconomic status [32], low-income areas [33, 34], poverty [35] and poor housing conditions [36].

The spread of dengue usually occurs in centrifugal waves as a pattern of expansion [35, 37]. Low-income clusters seem to play an important role in initiating the dispersion of dengue to adjoining territories in Vitória. Until December 2012, an intense transmission was practically restricted to the areas of the clusters 1 and 3. However, in January 2013, dengue dispersed to different areas with notable intensity in other low-income clusters. After April 2013, areas surrounding low-income clusters presented increased incidence, possibly influenced by their proximity to these places. The isolation of cluster 6, which is surrounded by the airport and the industrial park, seems to contribute to a distinct pattern of dengue occurrence across the time within its territory, without suffering influences of other populations. The relative isolation of the cluster 11 from other clusters seem to contribute to the delayed incidence increment. Therefore, living in high-income areas seems to contribute to a late increase in dengue incidence.

This study presents limitations inherent to its ecological approach, since the analyses are not based on individual, but aggregated data. In addition, the study utilized secondary data collected for surveillance purposes, thus absence of data on patient addresses resulted in the exclusion of 2.8% of the sampled population (n = 536). Dengue is a disease with mandatory reporting. However, due to its nonspecific signs and symptoms, dengue could be confounded with other diseases. This may lead to both over- and underreporting, depending on the degree of alertness towards dengue in the attending health care worker. In addition, dengue presents a high proportion of asymptomatic or subclinical cases, precluding the capture of the real number of incident infections, restricting the sample size to cases that sought treatment in health services. The study included also cases that were not confirmed by laboratory tests, thus, the presence of erroneous diagnoses of dengue could have occurred. Therefore, the inclusion of other DENV serotypes (1, 2 and 3) or other viral infections, including those transmitted by *A*. *aegypti* with dengue-like symptoms, could have occurred, despite the fact that the first identifications for example of Chikungunya virus and Zika virus in the city are dated from 2015. The use of MMQGIS for geocoding implies a certain degree of imprecision higher than when using GPS equipment, since the geocode by MMQGIS depends on algorithms and matching with the database of Google Maps ™ API, which can vary in quality across locations. Nevertheless, all geocoded points were checked individually in order to minimize non-matches. Among them, 99.5% were geocoded in street level and 0.5% in district level.

The study has a circumstantial advantage since the immune status of the population regarding DENV-4 protection did not influence the dengue distribution in the beginning of the epidemic, considering that the circulation of DENV-4 was unprecedented in the territory. The results also indicate areas to be prioritized in future preventive actions in urban areas facing introduction of a new DENV serotype. The use of geographic information systems should be encouraged in dengue surveillance, since knowledge on the distribution of cases at different time points not only favors immediate actions but also helps to predict spatial distribution of cases, since the dispersion is partially influenced by the immunity of local population and by the proximity to hotspots [38]. In a city with an unprecedented circulation of a certain DENV serotype and, consequently, a highly susceptible population, low-income areas seem to be more prone to suffer from intense transmission of dengue. Therefore, the identification of a new serotype in a city requires preventive actions prioritized in these areas. Thus, successful preventive actions could minimize dengue spread in low-income areas and its adjacencies, and decrease the magnitude of dengue dispersion.

## Supporting information

S1 File Dataset (XLSX)
